# Cemento-osseous dysplasia: a multi-centre analysis of surgical management

**DOI:** 10.1007/s10006-025-01394-8

**Published:** 2025-05-07

**Authors:** Fadi Titinchi, Naser Alturki, Jean Morkel, Salem Alkaabi, Kathryn Taylor

**Affiliations:** 1 Department of Maxillofacial and Oral Surgery, Faculty of Dentistry and WHO Collaborating Centre, Tygerberg Hospital, University of the Western Cape, Private Bag X1, Tygerberg Oral Health Centre Francie van Zijl Drive, Cape Town, 7505 South Africa; 2 Department of Oral and Craniofacial Health Sciences, University of Sharjah, Sharjah, United Arab Emirates; 3 Department of Oral and Maxillofacial Surgery/Oral Pathology, Amsterdam University Medical Centers and Academic Centre for Dentistry Amsterdam, Vrije Universiteit Amsterdam, Amsterdam Movement Sciences, Amsterdam, Netherlands; 4 Department of Oral and Maxillofacial Surgery, Fujairah Hospital, Emirates Health Services, Fujairah, United Arab Emirates; 5 School of Medicine and Dentistry, University of Central Lancashire, Preston, UK

**Keywords:** Cemento-osseous dysplasia, Fibro-osseous lesions, Maxilla, Mandible

## Abstract

**Purpose:**

Cemento-osseous dysplasia (COD) is a fibro-osseous lesion whose management is highly controversial in the literature. Due to scarcity of comprehensive studies on its management, the aim of this study was to analyse its management and develop a treatment protocol.

**Methods:**

A multi-centre retrospective cohort analysis was conducted at two tertiary referral hospitals on 124 patients diagnosed with COD from 2005 to 2023. Demographic, clinical, and radiological data were analysed and correlated with treatment methods. Post-operative complications such as osteomyelitis or pathological fracture were documented along with follow-up visits to evaluate the need for further treatment. Data was analysed using Student’s t-test and Fisher’s exact test. Statistical significance was set at *P* < 0.05.

**Results:**

The patients’ ages ranged from 22 to 78 years (mean: 48.5 years), with majority being females (90.4%) and of African descent (95.9%). Radiopaque CODs presented significantly higher rate of symptoms compared to radiolucent or mixed lesions (*p* = 0.02). The majority of incidental CODs were managed through observation (72%), while six incidental CODs underwent biopsy due to suspicion of more sinister lesions. Symptomatic lesions were mainly treated by curettage (29.7%) or local excision (48.6%), while only one symptomatic case was managed with observation and antibiotics (*p* = 0.0001).

**Conclusion:**

Biopsy of asymptomatic COD should only be reserved for cases with inconclusive clinico-pathological features. The decision to surgically treat COD should be based on the presence of symptoms and infection. Early curettage or excision of infected COD is the most effective approach to eradicate the disease and prevent progression into osteomyelitis.

**Clinical trial number:**

: Not applicable.

## Introduction

A benign fibro-osseous lesion is a non-neoplastic condition characterized by the replacement of normal bone with a fibrous connective tissue matrix that includes abnormal bone or cementum [1]. Cemento-osseous dysplasia (COD) is the most prevalent among the six primary categories of fibro-osseous lesions acknowledged in the 2022 World Health Organization (WHO) classification of head and neck tumors [2]. Although it is a non-neoplastic lesion occurring exclusively in the dento-alveolar regions of the jaws, recent study has detected pathogenic hotspot mutations in the RAS-MAPK signalling pathway in COD, including mutations in BRAF, HRAS, KRAS, NRAS, and FGFR3 [3].

The lesion is relatively common in middle aged females of African and Asian descent, with an age-adjusted prevalence rate of 5.5%. Numerous studies have reported a male to female ratio of 1:9 [4, 5]. The exact aetiology of this lesion remains unknown, but genetic and hormonal factors have been suggested. Some authors propose that it may be a reactive lesion arising from the periodontal ligament [4, 5].

COD is sub-classified into three different variants based on the anatomical location of the lesion and its distribution within the jaws: Periapical COD, which has a predilection for the apical region of the mandibular incisor teeth, Focal COD involving a single tooth in a quadrant, usually in the posterior mandible; and Florid COD, which is more extensive and generalized with multi-quadrant involvement and tends to be bilateral, symmetric and may involve both jaws [6].

The diagnosis of COD can often be reached based on clinical and radiographic features alone, without the need for pathological confirmation, however, the management of these lesions remains contentious due to the risks associated with biopsy, including potential complications such as osteomyelitis and injury to adjacent vital structures. Conversely, refraining from biopsy of suspicious lesions can result in misdiagnosis and disease progression [6].

In the literature, there is notable scarcity of large, detailed studies on the management of COD, particularly in regions such as Africa and Asia where the lesion is most prevalent. Additionally, conflicting evidence exists from studies conducted across Europe, America, Asia, and Africa [7]. Therefore, further investigations into the management of this relatively common fibro-osseous lesion of the jaws are warranted. The aim of this study is to analyse the management of COD and develop a treatment protocol.

## Methods

This was a multicentre retrospective descriptive cohort analysis of patients diagnosed with COD at two tertiary referral hospitals (Tygerberg and Mitchell's plain Hospitals) for the period from 2005 to 2023. Ethical clearance was obtained from the Biomedical Science Research Ethics Committee (reference: BM22/7/5) of the University of the Western Cape prior to conducting the study. Patients were included in the study if they had a confirmed diagnosis of COD based on clinico-pathological correlation. In addition, the patient’s records needed to be complete with demographic, clinical, radiographic and treatment (if any) details. Patients with inconclusive diagnosis were excluded.

Data collection included patient’s age, gender, and ethnicity. The presenting clinical features including pain and infection, were documented alongside symptom history, with inclusion of incidental and asymptomatic lesions. Lesion locations within the jaws were characterised as anterior or posterior or both, notes on bilateral lesions and those present in all four quadrants. For each patient, the type of COD and its association with simple bone cyst (SBC) were recorded.

The radiodensity of each lesion was recorded and correlated with symptoms and treatment received. The effects of the lesion on adjacent anatomical structures (adjacent teeth, inferior alveolar canal and/or maxillary antrum) was documented. The treatment methods employed were recorded including observation, excision, partial removal or resection. The occurrence of any complications such as osteomyelitis or pathological fracture was documented. Follow-up visits and serial post-operative radiographs were evaluated for evidence recurrences.

The data collected was analysed using Student’s t-test and Fisher’s exact test to identify correlations between various clinico-pathological parameters and treatment methods. GraphPad statistical software was utilized for the analysis, which included means, standard deviations, and percentages. Statistical significance was set at *p* < 0.05.

## Results

A total of 141 patients were diagnosed with COD during the study period. Of these, only 124 patients met the inclusion criteria; the rest were excluded due to inconclusive diagnosis or poor-quality imaging. Notably, COD was the most prevalent of the fibro-osseous lesions in this population throughout the study period.

### Clinico-pathological features

The ages of patients at the time of diagnosis with COD ranged from 22 to 78 years (mean: 48.5 years). The majority of patients diagnosed with COD (76.5%) were above 40 years of age. Females constituted the most of affected individuals (90.4%), predominantly of African descent (95.9%). A significant proportion of patients with COD (61.3%) exhibited symptoms such as pain, swelling, or infection. A considerable number of cases were observed in fully edentulous patients, where COD was exposed through the gingiva/oral mucosa (39.5%). Additionally, paraesthesia of the lower lip was reported in four patients (3.2%).

Seven lesions (5.6%) were associated with osteomyelitis of the mandible, and five patients presented with submandibular draining fistulas, a consequence of long-standing infected COD. The duration of reported symptoms ranged from 1 to 48 months (mean: 9.5 months) prior to presentation. The majority of symptomatic COD lesions were located in the posterior mandible (95.6%).

Most CODs presented in the mandible (75.8%), predominantly in the posterior regions (75.5%). Focal COD was the most prevalent type (46.7%), followed closely by florid COD (45.1%), and lastly periapical cemental dysplasia (8.2%). Florid COD presenting in both jaws were fairly common (44.6%). On panoramic radiographs, the majority of CODs exhibited mixed density (51%). The vast majority of the lesions were well-defined (93.5%), with an irregular shape (62.1%).

Most CODs did not displace adjacent anatomical structures, such as maxillary sinus or inferior alveolar canal (82.1%). Only two cases (1.6%) were associated with SBC, and one case was associated with an odontogenic keratocyst (OKC). One patient developed osteosarcoma in association with florid COD. Importantly, radiopaque COD lesions were significantly more likely to be symptomatic compared to radiolucent or mixed density lesions (*p* = 0.02). However, there was no statistical correlation between the type of COD and the presence of symptoms (Table 1).

**Table 1:** Analysis of different variables based on symptom presentation (*n* = 124)

	Symptomatic		Total	*P*-value
	Yes	No		
Site				*p* = 0.39
Mandible	53	41	94 (75.8%)	
Maxilla	4	1	5 (4%)	
Both jaws	17	8	25 (20.2%)	
Radio-density				*p* = 0.02
Lucent	1	8	9 (7.2%)	
Mixed	34	29	63 (50.9%)	
Opaque	39	13	52 (41.9%)	
Type of COD				*p* = 0.56
Periapical	2	8	10 (8.1%)	
Focal	35	23	58 (46.8%)	
Florid	37	19	56 (45.1%)	

### Management

**Fig. 1:**
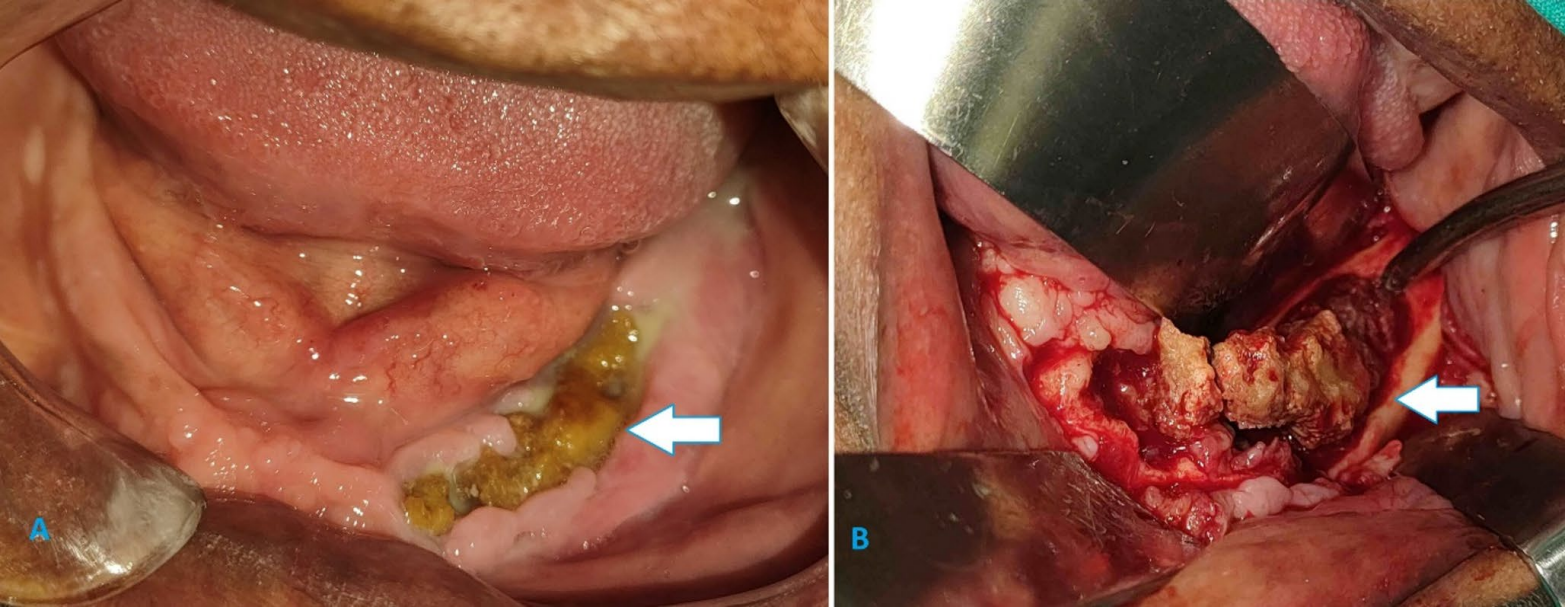
**(a)** Pre and **(b)** intra-operative findings of an infected COD in the left mandible

**Fig. 2:**
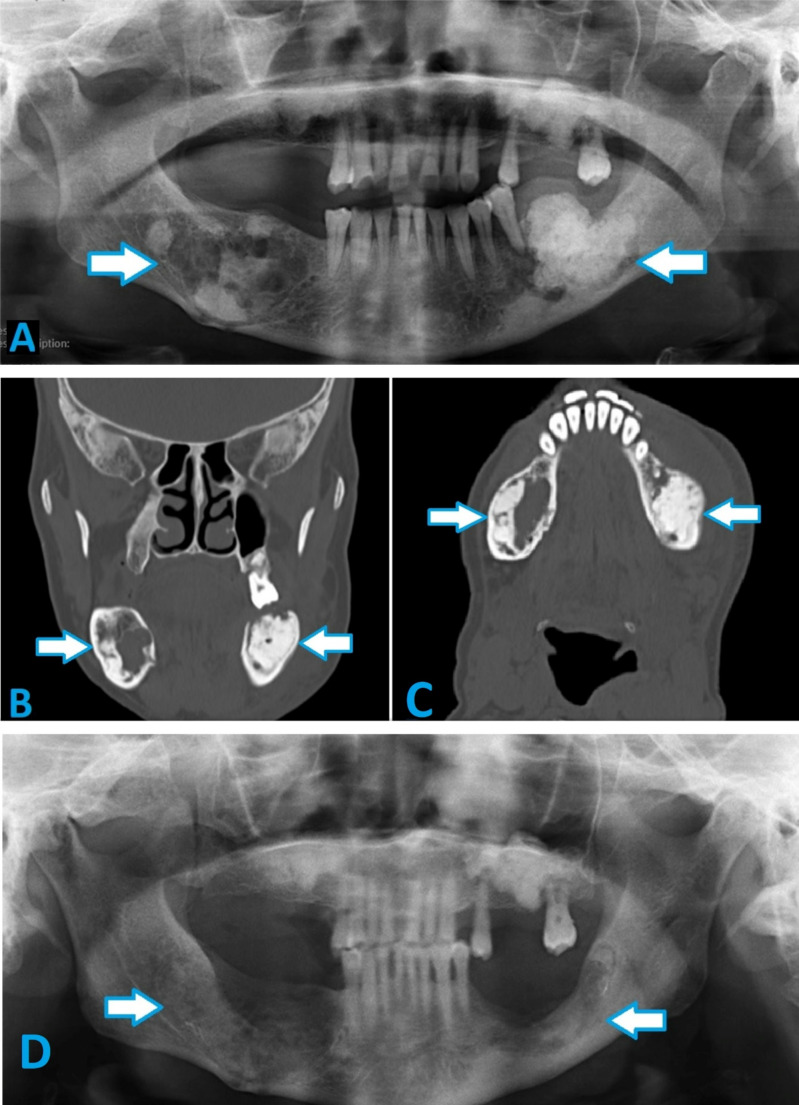
**(a)** Pre-operative panoramic radiograph of a patient diagnosed with florid COD complaining of pain and bilateral swelling in the mandible. **(b)** Pre-operative coronal and **(c)** axial CBCT images revealing expansive mixed density lesions in the bilateral mandible. **(d)** Seven months post-operative panoramic radiograph of the same patient showing good healing following excision of COD lesions from the posterior mandible

Symptomatic CODs were mostly managed with surgical removal, especially in cases that were secondarily infected (34.7%). When a fistula was present in association with infected COD, a fistulectomy was concurrently performed. Extensive lesions often required additional surgical debridement and sequestrectomy (Fig. 1), or radical resection in refractory cases. Partial removal or curettage was selectively performed for certain symptomatic lesions in order to avoid injury to adjacent anatomical structures (Fig. 2). Lesions were only biopsied if there was clinico-radiological concern that the lesion might mimic other sinister pathologies such as an osteosarcoma or odontogenic cyst or tumour.

In the analysis of the treatment methods, most radiopaque and mixed lesions underwent excision or curettage due to the increased risk of secondary infection following exposure to the oral cavity. A large number of mixed density lesions were observed while more than half of radiolucent lesions were also observed. Despite these treatment patterns, there was no statistically significant relationship among them (*P* > 0.05).

In cases of infected COD, these lesions could easily be curetted from the rest of the uninvolved bone. In certain cases, due to the extensive size of COD, the lesion needed to be removed in piecemeal. The medical management protocol for infected CODs involved the administration of empiric antibiotics such as Amoxicillin/clavulanic acid (Augmentin^®^) or Clindamycin. The antibiotic regimen was adjusted as necessary, based on the result of the microscopy, culture and sensitivity to target cultured bacteria. Residual cavities in the jaw were packed with bismuth iodoform paraffin paste (BIPP) impregnated gauze, which was serially removed over a period of 2–3 weeks.

The majority of incidentally found CODs were managed by observation and serial follow-up (72%), while six incidental CODs underwent biopsy due to clinical suspicion for other more sinister lesions. On the other hand, most symptomatic lesions were treated by curettage (29.7%) or local excision (48.6%), while only one symptomatic case was observed and treated with antibiotics (Table 2). These differences in management patterns were statistically significant (*p* = 0.0001).

**Table 2:** Management patterns of incidental and symptomatic cods

Treatment method	Incidental	Symptomatic	Total
Observation	36 (29%)	1 (0.8%)	37 (29.8%)
Incisional biopsy	6 (4.8%)	15 (12.2%)	21 (17%)
Complete excision	6 (4.8%)	36 (29%)	42 (33.8%)
Curettage	2 (1.6%)	22 (17.8%)	24 (19.4%)
Total	50 (40.3%)	74 (59.7%)	124

### Complications

**Fig. 3:**
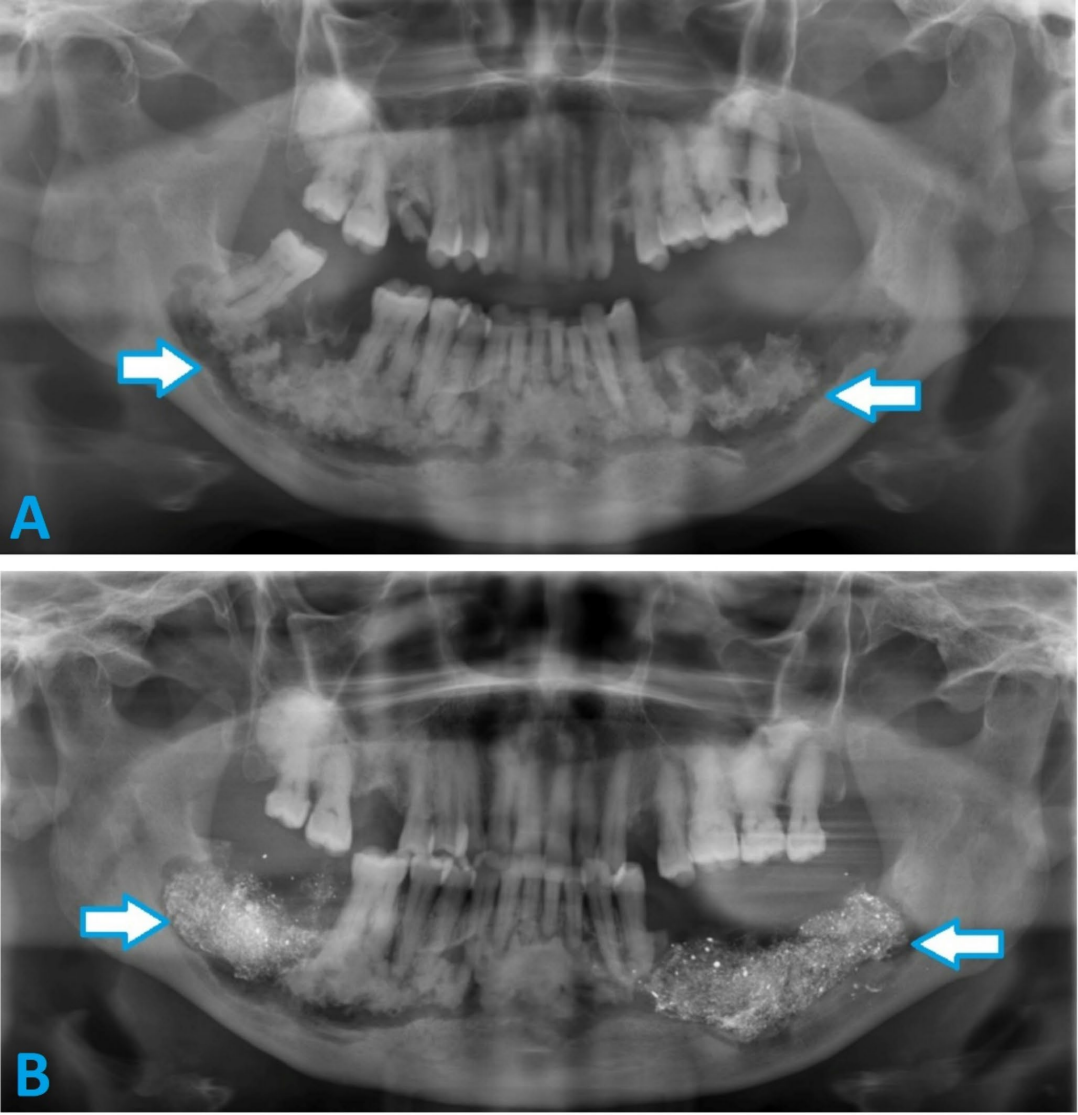
**(a)** Pre-operative panoramic radiograph of a patient with extensive florid COD and features of osteomyelitis in the mandible bilaterally. **(b)** Post-operative panoramic radiograph of the same patient post debridement of COD and placement of BIPP impregnated gauze

Within this sample, surgically treated lesions rarely required re-treatment due to secondary infection. The placement of BIPP impregnated gauze in the bony cavity after debridement reduced the risk of post-operative infection (Fig. 3). BIPP impregnated gauze also eliminated the dead space and circumvented the need for bone grafting thus reducing post-operative complications.

Three cases required re-operative surgery for removal of previously treated COD (mean follow-up: 7.9 months). One case of OKC associated with COD recurred four years post enucleation, likely due to the incomplete removal of the entire cyst lining, especially in areas closely associated with the COD. One edentulous patient experienced a pathological fracture of the mandible during surgical removal of COD. Extraction of teeth in close proximity to COD lesions was the leading cause of complications, which included secondary infection of COD, osteomyelitis, and development of cutaneous fistula.

## Discussion

Cemento-osseous dysplasia is a well described disease process whereby healthy bone is replaced by sclerotic bone. It is the most prevalent of the fibro-osseous lesions presenting in the jaws. Despite being fairly common in females of African and Asian origin, there is a lack of studies in the literature on COD in African populations [8]. Therefore, this study is one of the largest and most comprehensive on the management of COD in a predominantly African population.

### Clinico-pathological features

Middle-aged individuals of African ancestry are the population group most commonly affected by COD. The mean ages of patients diagnosed with COD in the literature ranges from 40.6 to 66.5 years, with an overall mean of 52.2 years [5–7, 9–14]. Females are significantly more likely to be affected with COD, whereas it is infrequently reported in males. This study’s findings align with these observations. The disproportionate prevalence of COD in females has been hypothesized to be due to hormonal imbalance that influence bone remodelling [15].

Generally, COD is described as an inconspicuous condition, usually detected incidentally on routine radiography. Some affected patients may present with swelling, pain and purulent discharge due to secondary infection in the bone surrounding the lesion. Studies in the literature report the presence of symptoms to range from 14.7 to 100% of cases [5, 7, 9–12]. In this study, 38.7% of cases were incidentally found on routine radiography.

There is consensus in the literature that the mandible is by far the most affected jaw, with the majority of lesions presenting in the posterior regions of the mandible [6, 7, 9, 10, 13, 14]. In current study’s sample, more than three quarters of CODs occurred in the mandible alone, while 20.2% occurred in both jaws. CODs occurring in the maxilla alone were uncommon.

There is conflicting data in the literature regarding which subtype of COD is the most common. Some studies report focal COD as the most prevalent subtype [6, 10, 14], while others identify florid COD as the most common [5, 7, 9, 11, 13]. In this study, focal and florid CODs were nearly equally common, with focal COD being slightly more prevalent. MacDonald-Jankowski [16] and Pereira et al. [6]. reported that all florid CODs involved the mandible, while nearly half also involving the maxilla as well.

The radiological features of COD are crucial for an accurate diagnosis. The majority of studies in the literature report that COD typically appears as a mixed density lesion on radiographs [6, 7, 9–11, 14]. However, studies focusing solely on florid COD have found that many lesions are radiopaque due to their mature nature [5]. In this study, nearly half of florid COD cases (46.4%) appeared radiopaque.

There is general agreement in the literature that CODs appear as well-defined lesions on radiographs in most cases [10]. Alsufyani and Lam [10] reported that 91.5% of CODs in their sample were well-defined. Similarly, in the present study 93.5% of lesions were well-defined. Ill-defined lesions warranted further investigations using advanced imaging or biopsy to rule out more sinister conditions.

In our study, the relationship of COD with adjacent anatomical structures was assessed using multiplanar CBCT/CT. Cortical bone aberration was the most commonly involved structure in most subtypes of COD, consistent with other studies [17]. Other frequently observed features included cortical bone thinning, expansion, and perforation. As previously reported, florid COD was responsible for most aberrations to the cortical bone compared to other subtypes of COD, further highlighting its aggressive nature [18, 19].

The accuracy of diagnosis of COD based on clinical and radiological examination is vitally important in order to avoid unnecessary biopsy of asymptomatic lesions. Although arriving at an accurate diagnosis may be challenging, it is essential for appropriate management. Ideally, COD should be diagnosed based on clinical and radiographic signs, with biopsies reserved for uncertain cases [6]. In this study, most cases were accurately identified as COD (sensitivity: 83.3%), a finding similar to other authors [10].

### Management

There is currently no consensus in the literature regarding when surgical treatment for COD is indicated and which method is most appropriate [7]. Generally, it is accepted that asymptomatic COD does not require surgical intervention beyond close clinical follow-up, preventive measures, and restorative methods to prevent the need for dental extraction and exposure of COD to the oral environment [9, 20].

The findings of this study underscore the necessity of biopsy in some asymptomatic cases to rule out other conditions. A biopsy is indicated if the diagnosis based on radiographic exam is uncertain, especially for lesions in the radiolucent phase or those causing significant bone expansion. Additionally, the presence of a second pathology, such as SBC, should prompt a biopsy to confirm the diagnosis [6]. This also applies to the rare occurrence of associated osteosarcoma, as observed in one of the cases in this study [21].

When COD lesions manifest symptoms, their management becomes more challenging due to development of chronic inflammation and bacterial infection within densely mineralized tissue. It has been described that there is poor penetration of antibiotics due to lack of tissue diffusion deeming them ineffective [6]. Consequently, performing a biopsy increases the risk of developing secondary infection, and removing of such lesions in asymptomatic patients can lead to significant morbidity [20].

Surgical debridement and curettage are recognised as the most appropriate methods for managing symptomatic and infected CODs (Table 3). Despite the challenges associated with treating infected lesions, particularly due to the poor vascularity of the area and increased bone hardness, these surgical techniques have shown effectiveness in treating the majority of infected CODs, with a success rate of 88.9% [7]. Antibiotics play an adjuvant role by inhibiting the formation of new biofilms through the destruction of bacterial colonies before they can adhere to the necrotic bone.

**Table 3:** Comparison of types of COD and their management in this study with other recent studies in the literature

	Type of COD			Treatment		
Author	Periapical	Focal	Florid	Observation	Biopsy	Curettage/Excision
Olgac et al. [6]	33 (24.4%)	83 (61.5%)	19 (14.1%)	None	135 (100%)	N/A
Pereira et al. [5]	None	None	82 (100%)	35	14 (34.1%)	
Kato et al. [7]	10.60%	27.30%	62.10%	N/A	N/A	59 (89.4%)
Elbeshir and Alhadad [9]	None	None	47 (100%)	17 (47%)	N/A	19 (40.4%)
Alsufyani and Lam [10]	93 (78.8%)		25 (21.5%)	N/A	N/A	N/A
Benaessa et al. [13]	16 (12%)	24 (18%)	93 (69.9%)	N/A	N/A	N/A
Nam et al. [14]	None	60 (96.7%)	2 (3.2%)	N/A	N/A	N/A
Decolibus et al. [11]	63 (33%)	43 (22.5%)	85 (44.5%)	N/A	73 (38.2%)	N/A
This study	10 (8.1%)	58 (46.8%)	56 (45.1%)	27 (29.8%)	21 (17%)	66 (53.2%)

*N/A: Not available

The primary prognostic indicator for the development of infected CODs in this study were identified as dental extraction and the use of removable dentures. Kato et al. [7]. reported comparable predictive factors for the onset of infection within COD lesions. Chronic secondarily infected COD were found to be more easily to curettage from the underlying bone, as the osteolytic process leads to the sequestration of the necrotic lesion. In these cases, patients generally exhibit compromised wound healing, precluding the application of bone grafting. Iodoform impregnated gauze has been shown to facilitate wound healing and the formation of granulation tissue. Certain authors have suggested the utilization of local soft tissue flaps, such as buccal fat pad, to aid in wound closure. Should there be a failure to heal after a period of 3 to 4 months, secondary surgical intervention should be considered [12].

Following an extensive literature search, very few studies focused on the management of COD. This study stands out as one of the most comprehensive analysis focused on the treatment of COD. Furthermore, using findings from this study along with data available from the literature, a management protocol is proposed (Table 4).

**Table 4:** Treatment protocol for Cemento-Osseous dysplasia

Treatment method	Indications
Observation	- Asymptomatic periapical, focal or florid COD with accurate diagnosis based on clinical and radiographic features - No exposure through oral tissues - No associated pathology with COD (such as OKC, SBC)
Incisional biopsy	- Clinical/radiographic diagnosis of COD is uncertain (e.g. radiolucent phase, extensive COD mimicking osteosarcoma) - Associated with another pathological lesion (OKC, SBC, osteosarcoma) - Lesion causing expansion
Debridement/curettage	- Symptomatic lesion closely associated with anatomical structures (inferior alveolar/mental nerve or maxillary sinus) - Lesion interferes with denture prosthesis
Local excision	- Symptomatic COD - Secondarily infected COD (with draining sinus/fistula or osteomyelitis) - Exposed COD through oral tissues

### Complications

One of the major complications associated with COD is the development of osteomyelitis, especially in the mandible, which leads to high morbidity for the patient and development of cutaneous fistulas, as seen in this sample. Elbeshir and Alhadad [9] reported that 25.5% of their Sudanese sample to have developed osteomyelitis, while Alsufyani and Lam ([0] reported an 11% prevalence. Kawai et al. [15]., in a Japanese study, reported a 14.8% prevalence, whereas in this study, only 5.6% of patients developed osteomyelitis. This lower rate of osteomyelitis in this study is likely due to aggressive and early surgical intervention in infected CODs cases to prevent progression to osteomyelitis. Elbeshir and Alhadad [9] managed most COD cases in their sample with antibiotics initially, while only refractory cases were treated with surgical debridement. This approach may explain the higher prevalence of osteomyelitis in their study when compared to this one.

The recurrence rate and need for surgical re-treatment of COD is another possible complication. Nam et al. [14]. reported excellent outcomes following surgical removal or curettage and administration of antibiotics, with no recurrence. In the current study, three cases (2.4%) required re-treatment due to incomplete removal. The decision for incomplete removal was initially made to avoid injury to vital structures, such as the inferior alveolar nerve.

Several limitations were encountered in this study, which are commonly encountered in other retrospective record-based studies. A significant number of records (approximately 12%) did not meet the study’s inclusion criteria due to missing information. For some older records, it was challenging to attain the patient’s clinical presentation due to incomplete record keeping. Given that the study was conducted over a period of 18 years and at two different hospitals, treatment methods could not be standardized, as multiple providers performed these procedures during this time period. Additionally, a significant number of patients were lost to follow-up, as many lived in rural communities far from these hospitals.

In conclusion, the decision to surgically manage COD lesions should be based on clinical signs and symptoms, as well as the presence of infection or osteomyelitis. Asymptomatic cases should be monitored periodically, with an emphasis on preventative measures to avoid secondary infections. Biopsy of asymptomatic lesions should only be performed when the clinico-pathological features are inconclusive and lesion could mimic other more sinister conditions. The presence of pain, suppuration and osteolytic changes with or without bone sequestration, are indications for surgical intervention in infected COD. Despite the lack of consensus regarding treatment regime, early curettage or surgical excision of infected COD is the most successful approach to eradicate the disease process and prevent further progression into osteomyelitis.

## Data Availability

No datasets were generated or analysed during the current study.
